# Rethinking the Architecture of Attachment: New Insights into the Role for Oxytocin Signaling

**DOI:** 10.1007/s42761-022-00142-5

**Published:** 2022-10-17

**Authors:** Kristen M. Berendzen, Devanand S. Manoli

**Affiliations:** 1 Department of Psychiatry and Behavioral Sciences, University of California, San Francisco, San Francisco, CA 95158 USA; 2 Center for Integrative Neuroscience, University of California, San Francisco, San Francisco, CA 95158 USA; 3 Weill Institute for Neurosciences, University of California, San Francisco, San Francisco, CA 95158 USA; 4 Kavli Institute for Fundamental Neuroscience, University of California, San Francisco, San Francisco, CA 95158 USA; 5 Neurosciences Graduate Program, University of California, San Francisco, San Francisco, CA 95158 USA

**Keywords:** Attachment, Oxytocin, Prairie vole, Neural circuit, Genetics

## Abstract

Social attachments, the enduring bonds between individuals and groups, are essential to health and well-being. The appropriate formation and maintenance of social relationships depend upon a number of affective processes, including stress regulation, motivation, reward, as well as reciprocal interactions necessary for evaluating the affective state of others. A genetic, molecular, and neural circuit level understanding of social attachments therefore provides a powerful substrate for probing the affective processes associated with social behaviors. Socially monogamous species form long-term pair bonds, allowing us to investigate the mechanisms underlying attachment. Now, molecular genetic tools permit manipulations in monogamous species. Studies using these tools reveal new insights into the genetic and neuroendocrine factors that design and control the neural architecture underlying attachment behavior. We focus this discussion on the prairie vole and oxytocinergic signaling in this and related species as a model of attachment behavior that has been studied in the context of genetic and pharmacological manipulations. We consider developmental processes that impact the demonstration of bonding behavior across genetic backgrounds, the modularity of mechanisms underlying bonding behaviors, and the distributed circuitry supporting these behaviors. Incorporating such theoretical considerations when interpreting reverse genetic studies in the context of the rich ethological and pharmacological data collected in monogamous species provides an important framework for studies of attachment behavior in both animal models and studies of human relationships.

Social attachments, or selective affiliations between individuals, play a central role at all levels of human relationships and represent a key determinant of psychological and physical health (Bowlby & Bowlby, 1982; Holt-Lunstad et al., 2010; O’Connor & Rutter, 2000; Robles et al., 2014; Rutter et al., 1999). Understanding social behavior in general, and attachment in particular, has been of intense interest for those studying affective biology and its neural substrates in recent years. The complexity of such behaviors presents a challenge to constructing cohesive theoretical and experimental frameworks that link observed behaviors with activity and molecular changes in the neural circuits underlying these behaviors (Adolphs, 2009; Anderson & Adolphs, 2014; Goodson, 2013). We aim to provide an introduction to select developmental and neurobiological concepts, namely developmental redundancy, modularity, and distributed circuitry, related to the regulation of attachment behaviors. While these concepts have been elegantly reviewed elsewhere (Anderson & Adolphs, 2014; Anderson, 2016; Hoke et al., 2019), we apply them to understanding the differential effects of specific genetic and neuroendocrine factors (oxytocinergic systems) on defined social attachment behaviors, which may apply not only in animal models but to human behavior as well.

Social attachment is defined by the emotional bonds formed between human infants or young non-human animals and a parent or caregiver as well as bonds between unrelated partners or peers in adulthood (Ainsworth, 1979; Bowlby & Bowlby, 1982; Harlow & Harlow, 1965). Across species, social attachments manifest in complex, but similar, patterns of behaviors, including mate (or pair) bonding, parental behavior, and kin and peer affiliation (Bales et al., 2017; Reichard & Boesch, 2003; Turner et al., 2010; Winslow, Shapiro et al., 1993). Socially monogamous species, representing ~4 to 9% of mammals, allow us to investigate the genetic and neurophysiological mechanisms mediating long-term attachments across the lifespan (Kleiman, 1977; Lukas & Clutton-Brock, 2013). Social attachments are organized around the formation and maintenance of social bonds. One of the most intriguing adult attachments is the enduring bond between mates. Pair bonds are characterized by long-term, preferential mating between two individuals and the active rejection of novel potential mates. Thus, pair bonding represents a rich substrate by which to begin to dissect the mechanisms that mediate and integrate socio-affective processing and behavior.

Robust animal models are essential for human-comparative analysis of attachment behaviors and identification of conserved molecular entry points into the neural circuits for pair bonding and associated affective processes. Studies of prairie voles (*Microtus ochrogaster*), a socially monogamous rodent species, have been foundational in our understanding of the biology of attachment. Prairie voles were first identified as living in burrows in extended families, and consistent male-female pairs are trapped together in the field (Getz et al., 1981). In the laboratory, prairie voles have been compared to closely related promiscuous species; such studies have provided a basis for understanding the behaviors associated with social monogamy. Prairie voles display long-term social attachments between mates (and peers), as mating partners show an enduring pair bond characterized by preference for spending time in close contact with a partner (Carter & Getz, 1993; DeVries et al., 1996; Getz et al., 1981). This “partner preference” has traditionally been tested in the laboratory using a preference assay, in which a bonded vole is given access to its bonded mate or a novel animal of the opposite sex (Beery, 2021; Carter & Getz, 1993). Bonded prairie voles will spend a majority of time with their partner in such a paradigm. The formation of affiliative bonds dramatically modifies patterns of other innate social behaviors such as aggression and mating, as bonded animals vigorously reject novel potential mates and avoid incest (Carter & Getz, 1993; Resendez et al., 2016; Resendez & Aragona, 2013). Such behaviors are displayed by both sexes and prairie voles show biparental care of offspring (Carter & Getz, 1993; DeVries et al., 1997). Furthermore, separation of bonded mates results in increased anxiety-type behaviors and physiological changes that accompany stress, supporting integrated neural and physiologic mechanisms that facilitate the preservation of such attachments within species (Grippo et al., 2007, 2011; Martin II et al., 2006; Resendez et al., 2016; Resendez & Aragona, 2013).

## Oxytocin and Vasopressin Signaling in Pair Bonding

The evolution of varied complex social systems and affiliative behaviors, including social attachment behavior, has intriguingly converged upon the nonapeptide hormones oxytocin (Oxt) and arginine vasopressin (AVP), and their homologues, despite arising in the context of diverse ecological pressures and social constraints (Bielsky & Young, 2004; Carter, 2017; Carter & Perkeybile, 2018; Donaldson & Young, 2008; Feldman, 2017; Insel et al., 1998; Opie et al., 2013; Reichard & Boesch, 2003). Following the initial establishment of Oxt function in the physiology surrounding parturition, namely uterine contraction and milk ejection, investigations of its role in maternal behaviors revealed that Oxtr signaling modulates a range of attachment behaviors across species (Lee et al., 2008; Nishimori et al., 1996; Pinto et al., 1967; Reynolds et al., 1950; Rich et al., 2014; Shapiro & Insel, 1992; Wakerley et al., 1973; Young et al., 1996). These include the initiation of maternal behavior in rodents as well as the quality of maternal-infant interactions in humans (Marlin et al., 2015; Meyer-Lindenberg et al., 2011; Pedersen et al., 1982; Strathearn, 2011). Such investigations eventually lead to its implication in pair bonding (Insel et al., 1998; Shapiro & Insel, 1992).

**Fig. 1:**
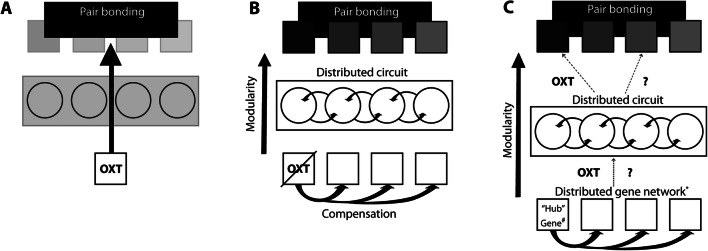
Models of oxytocin function within circuits encoding pair bond behavior. **A** Common model based on historical studies suggesting oxytocin acts as a primary genetic mediator of pair bonding behavior, whether through its action on local circuitry in select brain regions or on nodes integrated within a distributed circuitry. This is also in contrast to its potential modular action on specific subcomponents of pair bonding behavior, where other genes or gene networks may independently regulate distinct components of pair bonding (both shown in gray). **B** Model depicting three hypotheses: one in which the loss of Oxt/Oxtr is compensated for by other genes, another proposing a modular structure of genetic and neural circuit architecture, and finally, the potential for distributed but coordinated circuitry mediating distinct domains of pair bonding behavior (gray squares). **C** A third model depicting the hypotheses in **B**, but indicating a neural structure that is not specified by Oxt/Oxtr, but by either another regulatory “hub” gene (*#*) or a distributed gene network (*). In this model, Oxt signaling and/or other molecular mediators may act to control aspects of gene or circuit function (dashed lines) without being necessary for development of the underlying circuitry for pair bond behaviors. Parallel or compensatory processes may play a role in mediating specific behavioral outputs at any of the genetic or neural levels of regulation depicted

Elegant work applying behavioral, pharmacological, and viral approaches to understand attachments in prairie voles revealed a role for these same neuroendocrine mediators, (Carter et al., 2008; Cho et al., 1999; Insel et al., 1998; Insel & Hulihan, 1995; Keebaugh et al., 2015; Shapiro & Insel, 1992; Winslow, Hastings et al., 1993). Pioneering studies identified interspecies variation in the patterns of expression of Oxtr and the vasopressin 1a receptors (V1aR) that correlates with the potential for pair bonding between closely related vole species (Carter et al., 1995; Shapiro & Insel, 1992; Wang & Young, 1997; Winslow, Hastings et al., 1993, Winslow, Shapiro et al., 1993). Pharmacological inhibition of Oxt and AVP signaling, either applied systemically or localized to brain regions enriched for receptor binding in prairie voles, was sufficient to disrupt pair bonding following cohabitation. Consistently, exogenous administration of these hormones promotes pair bonding under conditions that do not typically result in pair bond formation (Carter et al., 2008; Cho et al., 1999; Insel & Hulihan, 1995; Lim & Young, 2004; Liu et al., 2001; Wang et al., 1998; Winslow, Hastings et al., 1993, Winslow, Shapiro et al., 1993). In line with these observations, viral manipulations that increase or decrease Oxtr expression in specific brain regions mirror pharmacologic agonism or antagonism of its signaling, respectively (Keebaugh et al., 2015; Keebaugh & Young, 2011; Ross et al., 2009). Such responses to manipulation suggested the primacy of oxytocin, and presumably its signaling via Oxtr, in mediating the formation and behavioral expression of partner preference (Keebaugh & Young, 2011; Numan & Young, 2016; Fig. 1A). However, our understanding of the genetic and neural control of attachment behaviors is incomplete. For example, the full extent of the neural circuits affected by Oxt modulation is not fully understood, nor are the compensatory contributions of the AVP system, the genetic regulation of Oxtr expression across development, and the action of Oxt at the synapse, among others. A large gap in our understanding of the genetic and neural substrates of attachment behaviors stems from a previous absence of tools to manipulate the prairie vole genome constitutively throughout development and with spatial and temporal control.

## Molecular Genetics Applied to Attachment Behavior

Modern molecular genetic tools will help clarify our understanding of how neuroendocrine factors mediate bonding and attachment behaviors and related affective states as we implement them in new species. Interference with a gene’s function is commonly used to elucidate its role in a biological process (Baker et al., 2001; Konopka & Benzer, 1971; Nüsslein-Volhard & Wieschaus, 1980; Vitaterna et al., 1994). Recent developments in the tools for generating such alterations to gene expression have made it possible to implement these techniques in a wide variety of species with high efficiency and relatively low barriers to entry. Such tools generally rely on knockdown, or the reduction in expression of a given gene, or knockout, the complete loss of expression of a targeted gene. Given the central role of oxytocin signaling in affiliative and parental behaviors across taxa, we and other groups adapted clustered regularly interspaced short palindromic repeats (CRISPR)/Cas9-based molecular genetic tools to generate prairie voles that lack the oxytocin receptor (Oxtr; Horie et al., 2019; Berendzen et al., 2022). We examined partner preference following cohabitation for one week with an opposite sex partner using the established assay described above (Beery, 2021; Williams et al., 1992). We made the surprising finding that Oxtr is genetically dispensable for pair bond formation, as partner preference was maintained in Oxtr knockout animals (Berendzen et al., 2022). Horie et al., 2019 examined a different subset of behaviors commonly attributed to oxytocinergic regulation, including pup vocalization, anxiety-like behavior, alloparental behavior (parental behavior exhibited by individuals towards non-descendant young), and sociability. They found no difference in Oxtr knockout animals compared to wild type in most of these behaviors, although did find mild increases in repetitive behavior, indicating anxiety-like behavioral differences, and impairment in preference for social novelty (Horie et al., 2019). These surprising findings suggest a differential requirement for oxytocin signaling across distinct domains of affective, attachment, and other social behaviors in prairie voles.

Pharmacological and viral-based interventions have been used to examine oxytocin signaling primarily in adulthood and have demonstrated a role for oxytocin in controlling pair bond formation. One initial interpretation of the differential results between pharmacological knockdown and constitutive genetic knockout is that the drugs themselves are non-specific and act at other sites to influence behavior (Busnelli et al., 2013; Manning et al., 2012). While promiscuous action of pharmacological agents should be considered and examined in interpreting these seemingly disparate results, other developmental and neurobiological mechanisms may be at play. Incorporating frameworks from developmental biology will aid in interpreting gene-modifying studies and broadly apply when considering directed molecular genetic approaches in the context of ethological and pharmacological studies of behavior.

Below, we discuss potential hypotheses derived from common concepts in the evolutionary-developmental literature for understanding the differential effects of constitutive, whole-organism, loss of function alleles of specific genes, such as in knockout models, in comparison to later manipulations of expression or activity in adulthood, as seen with knockdown of expression or pharmacological studies. We first address the developmental timing of intervention and the potential for invoking compensatory or parallel mechanisms with constitutive loss of Oxtr signaling, as compared to selective inhibition postnatally or in adulthood. Next, we consider the evidence for modularity in the genetic and neural systems underlying attachment behaviors, the study of which is facilitated by targeted genetic approaches. Finally, we examine the impact of constitutive deletion of the receptor in all tissues and cell types vs. regional or restricted depletion of Oxtr signaling, and discuss the differential impacts on activity across distributed circuits that may underlie pair bonding behaviors. For each discussion, we provide evidence from various species and systems, followed by support for similar processes in pair bond behavior and their potential regulation by oxytocinergic signaling. These proposed mechanisms are not mutually exclusive and any or all of these processes may be integrated at various levels to produce the pair bonded state and influence associated behaviors (Fig. 1B, C).

Importantly, the conceptual approaches we discuss may also have implications for our understanding of affective processing and the underlying developmental and neural circuit impacts on human attachment behavior. Patterns of relationships transform over the life course, and attachment with parental figures during early development may powerfully impact the quality and style of attachments in adulthood (Ainsworth, 1979; O’Connor & Rutter, 2000; Rutter et al., 1999). This suggests strong developmental regulation of the neural mechanisms underlying attachment behaviors. The formation and persistence of social bonds require coordination of a multitude of affective and cognitive domains, including social motivation, learning and memory, reward and valence processing, and threat detection. These domains are integrated to guide the display of prosocial and agonistic behaviors in the appropriate ethological context (Gustison & Phelps, 2022; Insel & Young, 2001; Krach, 2010; Resendez & Aragona, 2013). Each of these domains and related behaviors has been attributed to specific regions and circuits in the brain. Local neural circuits are also integrated into functionally connected networks that are interrelated and mediate multiple affective domains that influence attachment behaviors (Gustison & Phelps, 2022; Seeley et al., 2007). For example, activity in the amygdala is thought to encode valence, the positive or negative affective response to a stimuli, as well as other affective domains (Lanska, 2018; Tye, 2018). However, this area is part of a distributed network of anatomically and functionally connected neural circuits implicated in the processing of social information, e.g., the salience network (Seeley et al., 2007). Activity within and across these networks may contribute to both the development of positive valence associated with an attachment figure and the effects of healthy attachments on adaptive buffering of emotional response to strongly positively or negatively valenced stimuli (DeWall et al., 2012; Gillath et al., 2005; Kubzansky et al., 2009; Perry et al., 2017; Vrtička et al., 2012). While numerous theoretical frameworks have been applied to the understanding of human attachment and the underlying neurobiology, the application of concepts from fields like developmental and evolutionary biology will aid in deepening and testing our understanding of such models and the molecular, neural, behavioral, and psychological processes involved.

### Developmental Factors may Buffer Variation in Oxytocinergic Systems to Preserve Pair Bonding

Previous studies in prairie voles described above primarily employed experimental paradigms in which Oxtr signaling is manipulated in adulthood, immediately prior to specific social interactions or assays of behavior, but is present and active throughout development. Manipulations of Oxtr function in the wild-type adult brain may have distinct effects on attachment behaviors when compared to the constitutive, organismal absence of its function throughout development**.** This dichotomy is well articulated in developmental biology, whereby genetic contributions to behavior directly affect developmental processes, thus “organizing” or specifying the process. Alternatively, genetic manipulations may contribute to adult function of the gene product itself, having “activational” effects in adulthood (Baker et al., 2001; Hammock, 2014). Across species, compensatory mechanisms may thus arise in the context of genetic perturbations during development resulting in distinct phenotypes to those due to impairment of gene function later in life (Daude et al., 2012; De Souza et al., 2006; El-Brolosy & Stainier, 2017; Rossi et al., 2015; Smart & Riley, 2013; White et al., 2013).

*Canalization* is the tendency for development of a specific phenotype to follow the same trajectory under different conditions, such as different environments or different genetic backgrounds (Hoke et al., 2019; Waddington, 1959). The presence of mechanisms, whether compensatory for or parallel to Oxtr function, that support the preservation of pair bonding in voles provides evidence for the canalization of attachment behaviors in this species (Bergman & Siegal, 2003; Cadigan et al., 1994; El-Brolosy & Stainier, 2017; Rossi et al., 2015; Tautz, 1992; Teng et al., 2013). Canalization by mechanisms such as those detailed below may indicate strong evolutionary pressures to maintain certain behavioral phenotypes. The persistence of attachment behaviors in the absence of Oxtr function, despite their regulation by Oxtr signaling in the context of wild-type development, suggests a species-specific selection towards the transition to monogamous mating strategies (Waddington, 1959). This is consistent with observations that loss of function mutations in genes with pleiotropic, or diverse, functions nevertheless often result in largely normal anatomy and function (Waddington, 1942; Wagner et al., 1997). Oxt and Oxtr display an enormous degree of such pleiotropy, affecting not only aspects of social behavior and maternal physiology, but also having widespread effects on feeding behavior and peripheral autonomic physiology (Carter, 2014; Dölen, 2015; Jurek & Neumann, 2018; Lawson, 2017; McCormack et al., 2020, p. 20). A number of theoretical and experimental studies demonstrate that such functional pleiotropy may be buffered evolutionarily both by genetic *redundancy*, often accomplished through gene duplications, and/or changes to regulatory mechanisms controlling tissue and context-specific expression levels of the gene. Either of these processes may compensate for gene loss (Cadigan et al., 1994; Carroll et al., 2006; Kafri et al., 2009; Tautz, 1992). We first discuss evidence in other systems for differential compensatory responses to genetic manipulation and then apply these principles to the potential for developmental compensation in oxytocinergic systems and pair bonding.

Diverse species exhibit evidence for developmental compensation with genetic manipulation. In zebrafish, knockdown of genes associated with vascular function, for example *egl-7 *or* vegfaa,* results in severe vascular deficits, while constitutive loss (knockout) of these genes throughout development reveals developmental compensation, as no obvious phenotype is observed (Rossi et al., 2015). Further studies reveal upregulation of compensatory gene networks that buffer against deleterious mutations in *egl-7*, which is not observed after translational or transcriptional knockdown later in development (Rossi et al., 2015). Transcriptional and phenotypic comparisons of knockout vs. knockdown of genes involved in diverse physiological processes in mammals, including metabolism, neural function, and vascular development (*Ppara, PrP-like Sprn,* and *thymosin beta-4* respectively), also reveal distinct compensatory responses. Mice-bearing null mutations for these genes lack the phenotypic effects seen in the context of knockdown of gene expression, in otherwise wild-type animals (Daude et al., 2012; De Souza et al., 2006; Smart & Riley, 2013).

The abundance and dynamic nature of oxytocin peptide and receptor expression during early development suggests a possible role in organizing the neural circuits for certain behaviors (Hammock, 2014; Newmaster et al., 2020). Expression of Oxtr mRNA, measured by quantitative PCR, is seen in rats and mice by embryonic day 12 (Chen et al., 2000; Tamborski et al., 2016). Oxt continues to be expressed in the developing brain, and mice show a steady increase in oxytocinergic cells in the hypothalamus and amygdala at postnatal days 1–8. In prairie voles, the number of Oxt expressing cells steadily increases from postnatal day 1 to 21 in males and females (Vaidyanathan & Hammock, 2017; Yamamoto et al., 2004). Oxytocin and its receptor show dynamic, species-specific changes in expression over the course of embryonic development and early postnatal life, impacting the development of cortical and subcortical circuitry (Grinevich et al., 2015; Newmaster et al., 2020; Yamamoto et al., 2004). Developmental manipulations as well as regulation by gonadal hormones of oxytocinergic signaling have sex-specific effects on cellular expression of the peptide and receptor and on behavior in adulthood (Champagne et al., 2001; Hammock, 2014). In mice, early postnatal injection of Oxt leads to an increase in Oxt-expressing cells specifically in adult females (Yamamoto et al., 2004). In prairie voles, neonatal treatment with Oxt increases cFos immunoreactivity, a marker of neuronal activity, in Oxt-producing cells in male pups, while decreasing oxytocinergic signaling increased cFos in the same regions in females (Cushing et al., 2003). Interestingly, male prairie voles given a single perinatal injection of oxytocin showed increased partner preference in adulthood, while those given an oxytocin antagonist perinatally show no change from wild-type animals in partner preference during adulthood (K. Bales et al., 2004; Bales & Carter, 2003). Such time, dose- and sex-dependent effects on social behavior after exogenous manipulation of oxytocin suggests a high degree of plasticity in response to perturbations in oxytocinergic signaling during development.

While it is unknown what specific molecular and neural mechanisms facilitate attachment in the absence of Oxtr, the vasopressin neuropeptide system is an obvious candidate as an alternative and potentially compensatory mechanism of regulation on pair bonding behavior (Bosch & Neumann, 2008; Cho et al., 1999; Paré et al., 2016; Song et al., 2014; Yamamoto et al., 2004; Young et al., 1998). Evidence for overlap in these systems comes partially from evolution of the peptides themselves, which are homologs and likely resulted from a duplication of the gene for AVP (Borie et al., 2021; Grinevich et al., 2015; Theofanopoulou et al., 2021). It is also well-established that vasopressin receptors in multiple mammalian species bind Oxt, having clear functional consequences (Chini & Fanelli, 2000; Kelly & Goodson, 2014; Kesteren & Geraerts, 1998). Oxt displays high affinity for V1aR, and V1aR has been strongly implicated in mediating oxytocin driven effects on physiology and social behaviors (Chini & Fanelli, 2000; Everett et al., 2018; Stoop, 2012). For example, V1aR antagonists inhibit the ability of exogenous Oxt to induce flank marking, a form of social communication, in Syrian hamsters (Song et al., 2014). Additionally, in studies of methamphetamine seeking behavior in rats, co-administration of a V1aR antagonist significantly reduced the effects of Oxt on methamphetamine seeking, suggesting V1aR signaling mediates Oxt-dependent effects on reward behavior (Everett et al., 2018). Consistent with this model, paternal care by prairie voles is reduced only when both Oxt and AVP signaling are blocked (Bales et al., 2004). Studies aimed at rigorously determining changes in AVP or V1aR signaling in animals lacking Oxtr will likely elucidate if such mechanisms are at play. As an alternative to the evolution of compensatory mechanisms, parallel oxytocin-independent pathways may exist that support attachment behaviors and allow for the preservation of pair bonding despite absence of Oxtr function throughout development. In this case, Oxtr is not necessary for the development and patterning of the neural substrates for partner preference formation, but may later act on these substrates in a context and experience dependent manner to control their behavioral and affective output (Fig. 1C).

### Modularity of the Genetic and Neural Architecture Shapes Behaviors Supporting Pair Bonding

The selective nature of behavioral deficits with genetic loss of Oxtr suggests modularity in the genetic and neural encoding of pair bonding behaviors. In developmental terms, *modularity* can be defined as the division of a biological process into distinct units, each of which can develop or be regulated largely independent of other units (Hoke et al., 2019; Streelman et al., 2007; Xu et al., 2012). This concept has traditionally been applied to the action of genes during development to drive morphological phenotypes that are related but independently regulated by separable gene networks (Atchley & Hall, 1991; Hallgrímsson et al., 2002; Wagner, 2005). In this case, genetic modularity results in phenotypic modules. This concept can also be applied to understanding genetic regulation of behavioral phenotypes. As genes specify the development of local and interconnected neural circuits and thus influence the activity within these pathways, which in turn drives behavior, modularity is reflected at multiple neurobiological levels and in the behaviors that result. We present evidence for the modular structure of various social and non-social behaviors across species, and we discuss the potential for modular encoding of pair bond behaviors by oxytocinergic signaling (Greenwood et al., 2013; Levine et al., 2002; Weber et al., 2013; Weissbourd et al., 2021; Xu et al., 2012; Yang & Shah, 2014).

Some of the most striking evidence for modularity in behavior has come from studies of circadian behaviors as well as sexual behavior and associated sexually dimorphic regions within the brain (Anderson, 2016; Chan et al., 2002; Konopka & Benzer, 1971; Levine et al., 2002; Shah et al., 2004; Villella & Hall, 2008; Vitaterna et al., 1994; Xu et al., 2012). Studies in species ranging from fruit flies to mice have demonstrated that disruptions in individual genes that comprise the “molecular clock” result in specific changes in circadian patterns of behavior (Chan et al., 2002; Konopka & Benzer, 1971; Vitaterna et al., 1994). Similarly, studies examining sexually dimorphic gene expression in the hypothalamus and amygdala in mice identified sex-biased gene expression signatures in these regions. Targeted single gene disruptions within a subset of genes led to highly specific deficits in sex-typical behaviors while leaving other components intact, demonstrating separable genetic programs directing select behavioral subdomains (Xu et al., 2012). Such genetically separable modules have also been found to mediate social behaviors. Work in stickleback fish, *Astyanax*, examined schooling, a group behavior in which fish swim in a synchronized and polarized manner. Deconstruction of this dynamic social behavior identified separable genetic signatures that underly distinct components or modules of schooling behavior, such as tendency to school and body position (Greenwood et al., 2013). Studies of complex social behaviors like mating and aggression in fruit flies and mammals identified not only distinct genetically identified populations separably controlling these behaviors, but also distinct heterogenous neural populations in which both mating and aggression are influenced by functional changes in these neurons (Anderson, 2016; Asahina et al., 2014; Bayless et al., 2016; Certel et al., 2010; Koganezawa et al., 2016; Yang & Shah, 2014). These separable heterogenous populations may be functionally differentiated such that unique sets of genes regulate different neural modules and thus encode distinct behaviors reflecting this modular structure.

Studies in Oxtr knockout animals, including voles, mice, and rats, have demonstrated selective and species-specific deficits in behavioral and affective phenotypes suggesting modularity in the genetic and neural structures underlying attachment behaviors. Prairie voles bearing mutations in Oxtr demonstrated decreased affinity for social novelty in a three-chamber sociability test, without more general effects on sociality or anxiety-like behavior (Horie et al., 2019). Oxtr knockout mice and rats show decreased social recognition, without general deficits in sensory processing or generalized impairment of learning or memory, in addition to deficits in lactation and maternal nursing and reduced infant ultrasonic vocalizations (USVs; Numan, 1988; Pedersen et al., 1982; Takayanagi et al., 2005; Winslow & Insel, 2002). Oxtr knockout vole pups, in contrast, do not show decreased USVs when separated from parents (Horie et al., 2019). Disruption of species-specific subdomains of social and attachment behavior with loss of Oxtr suggests modularity in the pair bonding phenotypes regulated by Oxtr that may map onto specific neural circuit substrates. In Oxtr knockout mice, many brain regions, including the olfactory bulbs, lateral septum, piriform cortex, and dorsal lateral septum, show similar levels of activity (reflected by cFos induction) as wild-type animals after a brief social encounter, while less such activity is observed in the medial amygdala (MeA) and bed nucleus of the stria terminalis (BNST; Ferguson et al., 2001). The differential sensitivity across species in specific components of social behaviors, such as recognition, motivation or communication, to disruption of Oxtr may reflect not only separable genetic and neural modules, but differential selective pressure acting on these distinct modules in the evolution of social and attachment behaviors.

While further experiments are necessary to delineate the behavioral domains affected by loss of Oxtr and the genetic and neural substrates affected, examining social behaviors in the absence of Oxtr begins to reveal the modular structure and component behaviors that comprise attachment. The species-specific sensitivity of particular behaviors to loss of Oxtr signaling, and the potential selectivity of the brain regions involved provide further clues regarding the structural and functional building blocks of the systems supporting attachment behaviors. We can now combine molecular genetic techniques with, for example, transcriptomic and chromatin-profiling approaches and in vivo Ca2+ imaging to analyze gene expression and regulatory signatures, as well as patterns of neural activity across behavioral conditions and between species. These approaches will allow us to differentiate the genetically delineated components underlying pair bonding and their developmental origins (Gegenhuber et al., 2020). Understanding this modular structure may also have particular relevance for the integration of affective or internal states with sensory and environmental cues.

### Distributed Structure of Neural Circuits Affects the Demonstration of Pair Bonding

The striking observation that pair bonding occurs in the absence of Oxtr function may suggest that, like other neuromodulators, Oxt and Oxtr modulate specific behaviors not through binary, all-or-nothing action on isolated brain regions, but rather via coordinated, selective control of activity across a network comprised of multiple circuits. Such *distributed encoding* is observed in numerous neural systems underlying behavior across species, including olfactory processing, associative learning and motivated behavior, motor function, as well as mating and aggression and social behaviors more broadly (Anderson, 2016; Bargmann, 2012; Cachope et al., 2012; Cohn et al., 2015; Newman, 1999; Woolley et al., 2014; Yang & Shah, 2014). Complex behaviors may therefore be mediated by activity across a distributed network of circuits that each subserve distinct aspects of behavior, cognition, and affective components. We present evidence that neuromodulators, including Oxt, coordinate circuit activity across the network for a particular ethological context, developmental stage, and based on experience. We then discuss how complex attachment behaviors like pair bonding may similarly involve the coordination of a repertoire of closely interrelated, neuroendocrine-regulated circuits that mediate component behaviors.

Circuit organization characterized by parallel processing and functional “switches” between parallel streams allows for a distributed but interconnected circuit structure. Such a structure permits variability in specific components that comprise larger behavioral routines in response to genetic or environmental factors, and has been suggested as a mechanism by which the nervous system achieves behavioral flexibility (Bargmann, 2012; Chan et al., 2002; Falkner et al., 2014; Hashikawa et al., 2016; Mets et al., 2021; Ragozzino, 2007). Across such distributed networks, neuromodulators and hormones coordinate circuit engagement, i.e., levels or patterns of activity within local circuits or the influence of their output (Bargmann, 2012; Cohn et al., 2015; Marder, 2012). This is exemplified in the case of dopaminergic control of the neural pathways underlying motor behavior. Differential motor outputs are generated by functionally distinct parallel processing streams in the dorsal striatum. These streams, known as the direct and indirect pathways, are associated with specific neuronal populations defined by expression of distinct dopamine receptor subtypes. (Gittis & Kreitzer, 2012; Kravitz et al., 2010, 2012; Wiltschko et al., 2020). Dopaminergic signaling is also implicated in the reinforcement of specific aspects of animal behavior by signaling through distinct populations of cells in the basal ganglia (Graybiel et al., 1994; Kreitzer & Malenka, 2008; Tritsch & Sabatini, 2012). Similarly, in *Drosophila*, dopaminergic signaling in the mushroom body, a structure in the insect brain important for olfactory learning and memory, influences the flow of sensory information to direct specific circuit states that result in the appropriate enactment of innate and learned behaviors in response to olfactory cues (Aso et al., 2014; Cohn et al., 2015).

Studies across species suggest that oxytocin also functions as a neuromodulator across circuits recruited under specific ethological contexts. The invertebrate homologue of oxytocin, nematocin, coordinates circuit states in the context of mating behavior in the invertebrate species *C. elegans* (Garrison et al., 2012). Males lacking nematocin engage in mating attempts but exhibit fragmented sequencing of copulatory behaviors. This suggests that neuromodulator input across multiple local circuits involved in mating modulates the function and processing within these populations such that the outputs of these distributed networks are coordinated into coherent reproductive behavior (Bargmann, 2012; Garrison et al., 2012). That such functional compartmentalization of a distributed circuit occurs even in the absence of a centralized nervous system may reveal an organizing principle of neural circuits and systems. In rodents, the recruitment of specific circuits is mediated, in part, by the organization of Oxt production and function. Oxt is produced by two distinct cell types in the hypothalamus. These cells are anatomically segregated and project to distinct neural populations or release Oxt peripherally. The release of Oxt from multiple neuronal sites, including axons, soma, and dendrites, and its action on neuronal and non-neuronal cells throughout the brain and periphery may account for the functional division of behaviors and physiological processes linked to oxytocin signaling (Chini et al., 2017; Dölen, 2015; Dölen et al., 2013). Studies of long-range projections of oxytocin-producing neurons in the hypothalamus using mouse lines labeling oxytocin-expressing cells reveals widespread projection to diverse targets throughout the brain. Oxytocin fibers were identified in 29 brain regions in which Oxtr is expressed, including the islands of Calleja, frontal association cortex, shell of the nucleus accumbens (NAc), lateral septal nucleus, BNST, and MeA (Mitre et al., 2016). Furthermore, the correlation between fiber density and receptor expression is highly dynamic, beginning low in virgin females but increasing significantly in lactating females in both mice and rats, suggesting conserved, context-specific changes to circuitry through which oxytocin signals (Grinevich et al., 2016; Mitre et al., 2016).

Oxytocinergic signaling may act during pair bonding in prairie voles to coordinate the function of parallel and distributed circuits mediating bonding. Changes to activity across neurons projecting from the medial prefrontal cortex to the NAc within the context of pair bonding causally accelerates female affiliation towards naïve, opposite sex voles (Amadei et al., 2017). While the role for oxytocin in mediating the dynamic response of social behavior to corticostriatal activity is unknown, the coordination of activity across localized circuits to produce specific behavioral responses appropriate to distinct social settings and experience supports a role for context-specific control of distributed circuitry in mediating attachment behavior.

The nervous system thus balances developmental stability and flexibility to environmental stimuli using redundant or distributed processes comprised of genetically defined functional modules expressed in specific contexts (Cohn et al., 2015; Fig. 1B, C)*.* Local Oxtr signaling may modulate the behavioral expression of these modules only in the context of regional circuit activity that occurs during specific social behaviors (Dölen et al., 2013; Marlin et al., 2015). Alternatively, it might act to coordinate the activity across a subset of circuits and influence aspects such as the latency, likelihood, intensity, or duration of behaviors and associated affective states. In the case of systemic delivery of pharmacologic antagonists, differential or incomplete inhibition of signaling may disrupt coordinated activity to interfere with specific displays of behavior that manifest as global disruptions to pair bonding. Nevertheless, when development occurs in the absence of Oxtr, as with genetic knockout models, whether compensatory mechanisms exist or not, the innate coordinated activity of distributed circuits during salient social interactions, such as mating, may be sufficient to facilitate pair bonding. The transition in behavioral or affective states, manifested as attachment, may thus be encoded by an interaction between patterns of circuit activity and neuromodulation, not just by the modulators themselves.

## Conclusions and Future Directions

Numerous reviews and studies over the last decade have expressed caution in attributing widespread, functional necessity to oxytocinergic signaling in social behavior (Fink et al., 2006; Goodson, 2013; Guastella & Hickie, 2016). The availability of molecular genetic tools, in combination with pharmacologic and viral-mediated modulation, now allow us to genetically interrogate the neural and genetic substrates of attachment and other social behaviors with a new level of precision. We therefore believe that these results present an opportunity for reviewing and reconciling ideas about the modularity of genetic control of behaviors and the distributed function of neural circuits, providing a more comprehensive understanding of the role of oxytocin in social behavior and as an intervention in neuropsychiatric disorders of attachment.

The genetic and developmental perspectives offered here have implications for the intensive interest in and efforts to use oxytocin as a therapeutic agent for a host of clinical disorders in humans. Based on observations in prairie voles and other mammals including humans, clinical trials have sought to use exogenous Oxt and AVP or small molecule ligands to their receptors to ameliorate the deficits in social attachment and cognition experienced by patients with diverse neuropsychiatric conditions, yielding mixed results (Di Simplicio & Harmer, 2016; Feifel et al., 2010; Green & Hollander, 2010; Guastella & Hickie, 2016; Heinrichs et al., 2009; Hendaus et al., 2019; Leppanen et al., 2017; Parker et al., 2019; Rubin et al., 2010; Sikich et al., 2021). The initial pharmacological studies in voles and other species which sought to identify mechanisms underlying attachment behavior elucidated the first molecular candidates and their neural substrates. The advent of genetic and neural tools to monitor activity across distributed circuits in vivo, longitudinally label neural populations in response to activity, and capture the full range of molecular signatures associated with distinct behavioral states and defined cellular populations now facilitates an unbiased and comprehensive survey of the genetic, molecular, and neural landscape mediating attachment behavior.

The approaches to understanding neural mechanisms in behavior outlined here may also influence our understanding of affective processes that support attachment behaviors across species. Modularity in affective-related constructs like valence processing, reward, fear or stress response, and motivation, as well as a distributed circuitry across brain regions for encoding these affective states, is reflected in behavioral domains as well as endophenotypes within clinical syndromes (Anderson & Adolphs, 2014; Braff, 2015; Campbell, 2015; Jeste & Geschwind, 2014). Using the framework outlined, which strives to incorporate manipulations to behavioral systems at various developmental timepoints with context-specific perturbations to defined components of the circuitry, we can begin to address whether affective substrates become associated with behavioral states like pair bonding only with experience or whether they are fundamental to the developmental specification of attachment circuitry. Having genetic and neural entry points into the systems subserving attachment behaviors, derived from unbiased and comprehensive profiling of the molecular and circuit mediators of attachment behavior, will be essential as non-human studies are translated to work in humans, with careful consideration of the conservation of the structure and function of the systems and behaviors under examination.

## Additional Information

### Open Practices Statement:

(1) No additional data and/or materials exist for this manuscript.

(2) No experiments were conducted or preregistered.
